# Body mass index and subjective well-being in young adults: a twin population study

**DOI:** 10.1186/1471-2458-13-231

**Published:** 2013-03-16

**Authors:** Milla S Linna, Jaakko Kaprio, Anu Raevuori, Elina Sihvola, Anna Keski-Rahkonen, Aila Rissanen

**Affiliations:** 1 Hjelt Institute, Department of Public Health, University of Helsinki, Helsinki, Finland; 2 Institute for Molecular Medicine, Helsinki, Finland; 3 Department of Mental Health and Substance Abuse Services, National Institute for Health and Welfare, Helsinki, Finland; 4 Department of Adolescent Psychiatry, Helsinki University Central Hospital, Helsinki, Finland; 5 Department of Psychiatry, Helsinki University Central Hospital, Helsinki, Finland

**Keywords:** Body mass index, Subjective well-being, Life satisfaction, GHQ-20, Eating disorders, Twin study

## Abstract

**Background:**

Body mass index (BMI) is associated with subjective well-being. Higher BMI is believed to be related with lower well-being. However, the association may not be linear. Therefore, we investigated whether a nonlinear (U-shaped) trend would better describe this relationship, and whether eating disorders might account for the association in young adults.

**Methods:**

FinnTwin16 study evaluated multiple measures of subjective well-being, including life satisfaction, General Health Questionnaire (GHQ-20), satisfaction with leisure time, work, and family relationships, and satisfaction with sex life in young adulthood in the 1975–79 birth cohorts of Finnish twins (n=5240). We studied the relationship between indicators of subjective well-being and BMI both in full birth cohorts and in subgroups stratified by lifetime DSM-IV eating disorders.

**Results:**

We found an inverse U-shaped relationship between all indicators of subjective well-being and BMI in men. There was no overall association between BMI and subjective well-being in women. However, there was an inverse U-shaped relationship between BMI and indicators of subjective well-being in women with a lifetime eating disorder and their healthy female co-twins. Subjective well-being was optimal in the overweight category.

**Conclusions:**

Both underweight and obesity are associated with impaired subjective well-being in young men. The BMI reflecting optimal subjective well-being of young men may be higher than currently recognized. Categorization of body weight in terms of BMI may need to be reassessed in young men. BMI and subjective well-being are related in women with a lifetime eating disorder, but not in the general population of young women.

## Background

Body mass index (BMI) is associated with both physical and psychological health, including overall mortality, chronic somatic illnesses [1] as well as psychiatric disorders [2,3]. The relationship with BMI has been found to be U-shaped for mortality [4], depression [5], and quality of life [6-8].

While the link between obesity and impaired quality of life and lowered subjective well-being has been well documented [3,9-13], less is known about the relationship between subjective well-being and underweight. Low subjective well-being is related to low satisfaction in various life domains and high levels of psychological distress [14]. Low BMI and impaired well-being often coexist in young women [15,16], and severe underweight in young women is frequently caused by eating disorders. Anorexia nervosa (AN), bulimia nervosa (BN), and binge eating disorder (BED) affect at least 5.2-6.5% of young females at some point of their life [17-20], but the role of eating disorders in lowered well-being of young adults with extreme BMI remains unknown.

In this study, we studied the relationship between BMI and subjective well-being in a representative population-based sample of young adult twins. Given the high prevalence of eating disorders in this age group, we hypothesized that they could act as an important effect modifier of this relationship. To untangle the effects of eating disorders, we conducted stratified analyses among women with a lifetime eating disorder, their unaffected twin-sisters (representing those with a genetic susceptibility to eating disorders), and women who had neither had an eating disorder nor had a sister with an eating disorder.

## Methods

### The FinnTwin16 birth cohorts

FinnTwin16 is a nationwide longitudinal cohort study of health behaviors in twins and their families. Virtually all live twin births during 1975–1979 were identified in the central population registry of Finland. The twins were sent self-report questionnaires when they were 16, 17, 18 and 22–28 y, while their parents participated by answering questionnaires about themselves and the twins’ childhood at baseline. In our study we used data from the fourth wave of questionnaires (collected on each birth cohort semi-annually from beginning of 2000 to mid-2002). Data collection and analysis were approved by the ethics committee of the Department of Public Health, University of Helsinki and the Institutional Review Board of Indiana University, while psychiatric interviews of a subset were approved by the ethical committee of Helsinki and Uusimaa Hospital District. The interviewed subjects provided written informed consent, while the questionnaire study was accompanied with an extensive cover letter giving the purpose of the study and details of data protection. Subjects were informed that they could withdraw from the study at any time.

### Participants

FinnTwin16 wave 4 consists of self-reported data collected from 2415 males and 2825 females (response rates 78.8% and 90.0%, respectively). The participants were 22–28 years old (mean 24.5, SD 0.94) at the time of the study. The questionnaires assessed various health behaviors and included scales on subjective well-being (described below), as well as a screen on eating disorders, described in detail by Keski-Rahkonen et al. [21].

#### Exclusion criteria

We excluded subjects with any of the following conditions, which affect both body weight and subjective well-being: self-reported somatic illnesses (240 men, 276 women), pregnancy (118 women), self-reported lifetime psychosis or current antipsychotic medication (8 men, 7 women). It was not possible to control for depressive and anxiety disorder since the indicators used in our study are designed to measure mental distress and mood.

We excluded from analyses a total of 640 participants (248 men and 392 women) based on one or more exclusion criteria listed above. Weight or height values were missing for 16 men and 13 women (0.6% of the total sample). The sample used in the analyses consisted of 2151 men (out of whom 7 men with a lifetime eating disorder) and 2422 women (out of whom 2242 healthy women, 89 women with a lifetime eating disorder, and 32 healthy female co-twins of women with a lifetime eating disorder).

#### Female subgroups

Healthy women (n=2242) did not report psychopathology related to eating or weight. We invited women who screened positive for eating disorders (N=292) and their screen-negative female co-twins (N=134) to participate in diagnostic telephone interviews using the Structured Clinical Interview for DSM-IV (SCID; [22]) to obtain current and lifetime diagnoses of AN, BN, and BED (definition of BED according to DSM-IV research criteria). The diagnostic interviews are described elsewhere in detail [18,19,21]. The interviews identified 109 women with lifetime DSM-IV eating disorders. We excluded 20 of these women from the analyses according to the criteria described above. The final sample consisted of 89 women with eating disorders. We identified 52 female twin pairs discordant for DSM-IV eating disorders in our sample. Healthy co-twins did not report symptoms of eating disorders (n=32).

### Indicators of subjective well-being

We chose to assess subjective well-being using the following scales. Of these, GHQ-20 is designed for screening psychological distress in community settings and non-psychiatric clinical settings, such as primary care or general practice [23,24]. It is vastly studied, and shown to predict psychiatric morbidity. Life satisfaction scale is also a well-established measure. It is shown to correlate strongly with Beck Depression Index [25] and predict i.a. the risk of suicide [26]. In addition, we identified items in the questionnaire that had face validity in assessing subjective well-being; these included satisfaction with specific life domains.

#### Life satisfaction

Allardt’s four item scale life satisfaction (1973) measures levels of interest, happiness, easiness and loneliness of life [27]. The response alternatives were scored on a scale from 1 to 5, yielding a range of 4–20. Higher scores indicate higher dissatisfaction in life. Reliability of the scale was good (Cronbach’s alpha 0.71).

#### GHQ-20

General Health Questionnaire (GHQ-20) is a 20-item scale with a sum score range from 20 to 80, higher scores indicating an elevated level of psychological distress. GHQ-20 had excellent reliability in our sample (Cronbach’s alpha 0.91).

#### Satisfaction with leisure time, work, and family relationships

In the questionnaire, subjects assessed their satisfaction with leisure time at home, leisure time spent outside home, success at work or studies, and relationship with their co-twin, mother, father, and with their partner. As these items correlated highly, we used their sum score as a single variable in our analyses, with higher scores indicating higher levels of dissatisfaction. Cronbach’s alpha was 0.70.

#### Satisfaction with sex life

In the questionnaire, subjects assessed their satisfaction with sex life using a 5-point Likert scale, with higher scores indicating higher dissatisfaction.

*Body Mass Index* (BMI, kg/m^2^) was calculated from self-reported height and weight. The correlation of measured and self-reported BMI was 0.89, and the means of self-reported and measured BMI differed by 0.93 (95% confidence interval [CI] 0.79–1.07) kg/m^2^ in a subset of the cohort (n=566) [28]. For descriptive purpose we classified persons with BMI < 18.5 kg/m^2^ as underweight and those with BMI ≥30 kg/m^2^ as obese. We used sex-specific Z-score of BMI in all analyses.

### Covariates

In regression models, educational level, physical activity index, smoking status, total amount of consumed alcohol per month and drinking to intoxication were set as covariates. Educational level was a four-level categorical variable (compulsory school, vocational secondary education, academic secondary education, and tertiary education) [29]. We calculated physical activity index from the product of self-reported exercise intensity, duration (hours) and yearly frequency (days) as described by Mustelin et al. [28]. Smoking status had three categories based on the self-reported frequency of tobacco consumption: non-smoker (combining never and former smoker), light smoker, and heavy smoker (10 or more cigarettes per day). We used self-reported total amount of alcohol consumed during one month’s period and frequency of drinking until intoxication as covariates, as the total amount of consumed alcohol per time unit has been associated with body weight in many but not all studies [30,31] and drinking to intoxication and alcoholism are associated with psychological health [32].

### Statistical analysis

We conducted statistical analyses using Stata 11.0 software. The U-shaped association between BMI and indicators of subjective well-being was studied using linear regression modeling. We fit initially models with both a linear and a quadratic term for BMI. If the quadratic term (BMI-squared) was non-significant, we examined the significance of the linear association. We proceeded with two sets of analyses in two phases, the first set without adjustments and the second set adjusted for the covariates listed above. We used the whole sample to test the association between BMI and subjective well-being in the whole population. Next we applied the same regression models separately for healthy women, women with a lifetime eating disorder and their healthy female co-twins, in order to test the hypothesis that eating disorders or a familial predisposition to such disorders are an effect modifier for the relationship between BMI and subjective well-being. Comparison of the two latter groups indicated whether the association in women with eating disorders was attributable to the disorder or to an underlying familial, possibly genetic susceptibility. All analyses took clustering of twin individuals within pairs into account, as the subjects had been sampled as members of twin pairs [33].

## Results

### Descriptive statistics

Men had a mean BMI of 23.9 (SD 3.1) kg/m^2^ and women a mean BMI of 22.2 (3.5) kg/m^2^ (Table 1). Few men (1.2%) were underweight, while 7% of women were underweight (BMI <18.5 kg/m^2^). Obesity was relatively rare among these young adults, as approximately 4% of both men and women were obese (BMI ≥30 kg/m^2^) (for distribution of BMI see Additional file 1: Figure S1).

**Table 1:** Distribution of indicators of subjective well-being by categories of body mass index (BMI)

		**Number**	**Body mass index, BMI (kg/m**^2^**)**	**Minimum BMI at current height (kg/m**^2^**)**	**Life satisfaction **^**1**^	**General Health Questionnaire, GHQ-20 **^**1**^	**Satisfaction with leisure time, work and family relationships **^**1**^	**Satisfaction with sex life **^**1**^
			**Mean (SD)**	**Mean (SD)**	**Mean (SD)**	**Mean (SD)**	**Mean (SD)**	**Mean (SD)**
Men	All	2151	23.9 (3.1)	21.7 (2.3)	8.4 (3.0) ^2^	35.9 (7.4) ^2^	11.4 (4.3) ^2^	2.2 (1.0) ^2^
	BMI <18.5	26	17.9 (0.5)	17.1 (0.7)	8.8 (3.0)	37.8 (10.5)	12.5 (4.2)	2.0 (1.1)
	BMI 18.5-24.9	1496	22.4 (1.6)	20.9 (1.6)	8.5 (3.0)	36.1 (7.5)	11.4 (4.2)	2.2 (1.1)
	BMI 25–29.9	542	26.8 (1.4)	23.5 (1.8)	8.2 (3.0)	35.4 (6.9)	11.3 (4.3)	2.1 (1.0)
	BMI ≥30	87	32.6 (2.8)	25.4 (2.9)	8.3 (2.8)	35.6 (7.2)	11.2 (3.8)	2.4 (1.2)
Women	All	2422	22.2 (3.5)	20.0 (2.8)	8.6 (3.0) ^3^	38.8 (9.0) ^3^	11.6 (4.2) ^3^	2.1 (1.0) ^3^
	BMI <18.5	169	17.8 (0.5)	16.9 (0.9)	8.3 (3.1)	38.6 (8.6)	11.4 (4.2)	2.0 (0.9)
	BMI 18.5-24.9	1897	21.4 (1.7)	19.5 (1.9)	8.5 (3.0)	38.6 (9.0)	11.5 (4.1)	2.1 (1.0)
	BMI 25–29.9	261	26.9 (1.4)	22.6 (2.4)	9.0 (2.9)	39.5 (9.2)	12.0 (4.5)	2.1 (1.0)
	BMI ≥30	95	33.6 (3.4)	26.3 (5.1)	9.0 (3.5)	39.6 (9.0)	12.6 (4.6)	2.2 (1.0)
Female subgroups	Healthy women^4^	2242	22.2 (3.5)	20.0 (2.7)	8.5 (3.0)	38.5 (8.7)	11.5 (4.2)	2.1 (0.9)
	Women with a lifetime eating disorder ^5^	89	22.7 (3.8)	18.8 (3.3)	9.2 (2.8)	41.7 (10.9)	12.4 (4.5)	2.1 (1.1)
	Healthy co-twins	32	21.9 (2.4)	19.6 (1.8)	9.1 (2.5)	39.5 (9.4)	13.1 (3.5)	2.3 (1.2)

^1^ Higher scores indicate lower levels of well-being.

^2^ Skewness and kurtosis 1.0 and 3.9 for life satisfaction, 1.7 and 7.5 for GHQ-20, 0.5 and 3.2 for satisfaction with leisure time, work and family relationship, and 0.8 and 3.2 for satisfaction with sex life in men.

^3^ Skewness and kurtosis 0.9 and 3.5 for life satisfaction, 1.1 and 4.4 for GHQ-20, 0.6 and 3.3 for satisfaction with leisure time, work and family relationship, and 0.9 and 3.7 for satisfaction with sex life in women.

^4^ Healthy women were defined as not having a history of eating disorders or a twin-sister with a history of eating disorders. Women reporting any psychopathology regarding eating or weight have been excluded.

^5^ A lifetime eating disorder has been defined as having ever fulfilled the diagnostic criteria of DSM-IV anorexia nervosa, bulimia nervosa or binge-eating disorder.

Dieting behavior was common: 42.0% of women and 24.4% of men reported intentionally having lost weight more than 5 kg at least once. Current mean BMI was similar in healthy women, in women with a lifetime diagnosis of eating disorder, and in their healthy twin-sisters (Table 1). However, there were considerable differences between the weights of women with different eating disorder diagnoses. Divided by eating disorder diagnosis, the mean BMI was 21.2 (2.4) kg/m^2^ in the 32 women with AN, 23.6 (4.3) kg/m^2^ in the 37 women with BN, 20.1 (1.2) kg/m^2^ in the 9 women with AN and BN, and 26.2 (3.7) kg/m^2^ in the 11 women with BED (Table 2).

**Table 2:** Distribution of indicators of subjective well-being by lifetime eating disorder diagnosis

**DSM-IV Diagnosis**	**Number**	**BMI (kg/m**^2^**)**	**BMI <18.5 (kg/m**^2^**)**	**BMI ≥30 (kg/m**^2^**)**	**Minimum BMI at current height (kg/m**^2^**)**	**Life satisfaction**	**GHQ-20**	**Satisfaction with leisure time, work and family relationships**	**Satisfaction with sex life**
		**Mean (SD)**	**%**	**%**	**Mean (SD)**	**Mean (SD)**	**Mean (SD)**	**Mean (SD)**	**Mean (SD)**
Anorexia nervosa	32	21.2 (2.4)	9.4	0	16.5 (1.9)	9.6 (3.2)	40.0 (9.1)	11.7 (4.0)	2.2 (1.2)
Bulimia nervosa	37	23.6 (4.3)	0	5.4	20.4 (3.2)	8.8 (2.5)	41.9 (11.0)	12.3 (5.0)	2.0 (1.0)
Anorexia and bulimia nervosa	9	20.1 (1.2)	11.1	0	16.6 (2.3)	10.0 (2.8)	44.9 (16.0)	15.3 (2.2)	2.6 (1.3)
Binge-eating disorder	11	26.2 (3.7)	0	9.1	21.5 (2.3)	8.6 (2.9)	43.1 (11.4)	12.5 (5.5)	1.7 (0.9)

### Indicators of subjective well-being

Overall, women reported lower levels of subjective well-being than men (higher scores in life satisfaction, GHQ-20 and satisfaction with leisure time, work, and family relationships). Lean men tended to experience lower levels of subjective well-being than obese men, yet this could not be attributed to diagnosed eating disorders (five men had lifetime AN and two had lifetime BED). The opposite was seen among women, as obese women experienced lower levels of subjective well-being than lean women. Women with a lifetime eating disorder and their healthy twin-sisters reported lower levels of subjective well-being than other women (Table 1). Subjective well-being tended to be lower among women with eating disorders, with remarkably high distress expressed by women with lifetime diagnoses of both AN and BN (Table 2).

### Association of BMI with indicators of subjective well-being

#### Men

**Figure 1:**
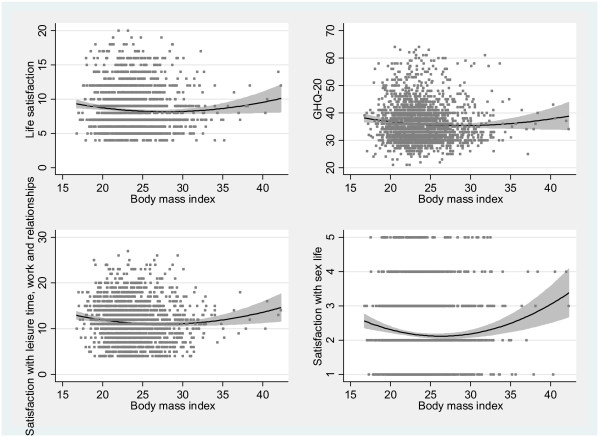
The U-shaped relationship between BMI and life satisfaction (p=0.003), GHQ-20 (p=0.005), satisfaction with leisure time, work, and family relationships (p<0.001), and satisfaction with sex life (p<0.001) in men.

All markers of subjective well-being correlated with BMI in both unadjusted and covariate-adjusted models. The adjusted quadratic regression model showed a U-shaped association curve with life satisfaction (p=0.003) and GHQ-20 (p=0.005), as well as with satisfaction with leisure time, work, and family relationships (p<0.001) and satisfaction with sex life (p<0.001) (Figure 1). The nadir of the U-shaped regression curve for all indicators of subjective well-being varied from +0.72 to +1.59 Z-score, corresponding to BMI of 26.1 to 28.9 kg/m^2^. Given the observed quadratic relationships, linear associations were not studied in men.

#### Women

The analyses were first done for all women and healthy women, both unadjusted and adjusted for covariates.

No statistically significant U-shaped relationships between BMI and subjective well-being were found in the whole group of women or healthy women (Table 3). However, in linear models, there was a trend toward association between BMI and dissatisfaction with leisure time, work, and family relationships (r=0.088, p=0.059 in the adjusted model). Also, there was a positive linear association between BMI and dissatisfaction with sex life (r=0.057, p= 0.007), which persisted also when the analyses were confined to healthy women (i.e. excluding women with eating disorders and their twin sisters) (r= 0.052, p= 0.016). No other linear associations were found.

**Table 3:** Linear regression statistics for the U-shaped adjusted association between Z-score of BMI and indicators of subjective well-being in all women (n=2422), healthy women (n=2242), women with a lifetime DSM-IV eating disorder (n=89), and healthy female co-twins of women with a lifetime eating disorder (n=32)

**Indicator of subjective well-being**	**Female subgroup**	**&Beta;-coefficient for the quadratic BMI term**	**Standard error**	**P**
Life satisfaction	All women	0.0456	0.037	0.20
	Healthy women	0.0332	0.042	0.43
	Women with a lifetime eating disorder	0.226	0.091	0.015
	Healthy co-twins	1.58	0.75	0.044
GHQ-20	All women	0.108	0.11	0.30
	Healthy women	0.0554	0.11	0.60
	Women with a lifetime eating disorder	1.46	0.40	<0.001
	Healthy co-twins	4.95	2.6	0.066
Satisfaction with leisure time, work, and family relationships	All women	0.0879	0.047	0.060
	Healthy women	0.0597	0.052	0.26
	Women with a lifetime eating disorder	0.554	0.17	0.002
	Healthy co-twins	0.591	1.1	0.58
Satisfaction with sex life	All women	0.0112	0.010	0.30
	Healthy women	0.0078	0.010	0.44
	Women with a lifetime eating disorder	0.0223	0.033	0.50
	Healthy co-twins	0.273	0.37	0.47

We report the statistics of the quadratic term (BMI-squared).

**Figure 2:**
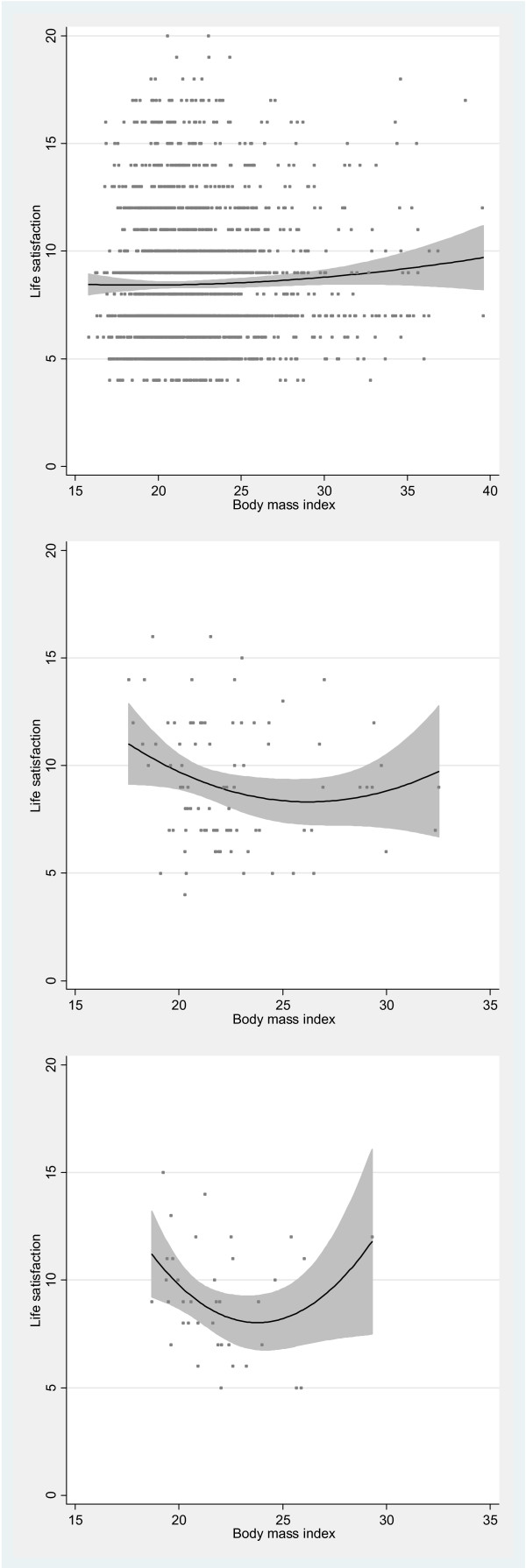
The U-shaped relationship between BMI and life satisfaction in healthy women (p=0.43), women with a lifetime eating disorder (p=0.015), and their healthy female co-twins (p=0.044).

Among women with lifetime DSM-IV eating disorders, a U-shaped relationship existed between BMI and life satisfaction (p=0.015), GHQ-20 (p<0.001). Same applied to satisfaction with leisure time, work, and family relationships (p=0.002) (Figure 2). The nadir of these regression curves was in the overweight category, ranging from Z-score +1.22 to +1.96, corresponding to BMI of 26.4 to 29.0 kg/m^2^. After excluding women with BED, the nadir of the regression curves ranged from +1.43 to +2.15 Z-score (BMI 27.2 to 29.7 kg/m^2^). Among healthy co-twins of women with a lifetime eating disorder, a U-shaped relationship existed between BMI and life satisfaction (p=0.044) and, respectively, a trend towards relationship between BMI and GHQ-20 (p=0.066) was observed. The nadir points for these regression curves were +0.41 and +0.59 Z-score (BMI 23.6 and 24.3 kg/m^2^), respectively. Table 3 shows the results of the analyses in all women and across the three studied female subgroups (i.e. healthy women, women with a lifetime eating disorder, and healthy female co-twins).

## Discussion

In our study, we found an inverse U-shaped relationship between BMI and subjective well-being in men. This relationship was constant across all indicators of psychological health, with highest levels of subjective well-being in the overweight category (according to World Health Organization classification of BMI, 1995). In women, BMI and subjective well-being were not associated. However, in females current or earlier eating disorder modified this relationship, and a similar effect modification was also seen among healthy twin-sisters of women with a lifetime eating disorder.

Our findings in men support those of previous studies on the relationship between BMI and subjective well-being in males. A positive correlation between obesity and impaired mental health has been identified in many studies [3,13,34,35], while the opposite has been reported in a few studies [36,37]. The effect of overweight on mental health remains controversial. A study on 43,534 Dutch adults [5] showed a U-shaped curve between depression and BMI, with least depressive symptoms in the overweight category. In the Canadian CaM*os* study on 9,423 adults, having weight within the overweight category was associated with slightly better health-related quality of life (HRQOL) in men [8]. In our study, estimates of the optimal BMI in terms of subjective well-being in men varied from 26.1 to 28.9 kg/m^2^.

Having one’s weight in the overweight category might even be beneficial for young men’s psychological health. This is probably in part attributable to the fact that men usually have higher lean body mass compared to women. BMI does not differentiate between fat and muscle tissue, thus BMI in the overweight range does not necessarily imply an excess of fat tissue. The highest levels of physical activity in men were seen at a BMI of 26.5 kg/m^2^ in this study (Additional file 2: Figure S2). In addition, young males’ body ideal is known to base largely on muscularity, and muscle dissatisfaction in turn is shown to be associated with psychological distress in the same FinnTwin16 cohorts [38] as used in the current study. Another possible explanation may lie in exposure to obesogenic environment; maintaining normal weight in such environment might be more stressful than allowing some weight gain, which in turn would result in optimal well-being in the overweight category in men.

In keeping with our findings, underweight has been related with impaired quality of life in men [39,40]. Low subjective well-being among men with low BMI in our study could not be attributed to a diagnosed eating disorder, but the effect of subclinical disordered eating and self-reporting bias cannot be excluded.

In studies of BMI and well-being, the authors have reported either an inverse U-shaped relationship [6,35], or a negative linear relationship [41] in women. While normal weight appeared to be optimal in most studies [6,35], an Australian study reported significantly lower psychological distress in overweight compared to normal weight female participants [36]. A limitation of these studies is that they did not take into account eating disorders, which affect both psychological well-being and body weight, and contribute to underweight in young adult women. In our study, the group means of most indicators of subjective well-being were highest among women with normal weight and lowest among obese women, but the differences were not statistically significant.

The relationship between BMI and subjective well-being was somewhat different in women with a lifetime eating disorder. The optimal BMI for subjective well-being appeared to be in the overweight range (BMI 26.4 to 29.0 kg/m^2^), also after excluding women with BED from the analyses. To our knowledge, the relationship of BMI and psychological health has not been studied earlier in women with a lifetime eating disorder. Due to the cross-sectional nature of our study, we were unable to determine how the altered relationship between BMI and subjective well-being develops as a function of time after onset or recovery. Interestingly, a similar effect modification was observed also in the twin-sisters of women with a lifetime eating disorder who did not report any psychopathology related to eating or weight. This suggests an underlying familial, possibly genetic susceptibility as an effect modifier of the relationship between BMI and subjective well-being. A previous study suggested shared genetic risk for depression and obesity as an explanatory factor for the association between these two conditions in women [42]. The findings on shared genetic transmission of depression and eating disorders have been ambiguous [43,44]. Our findings suggest that the relationship between BMI and subjective well-being is greatly attributable to a susceptibility to eating disorders in women. This might imply that body weight plays a greater role in the subjective well-being of these women compared to women without a susceptibility to eating disorders. Another explanation would be that when being exposed to mental distress, these women more readily react by either losing or gaining weight.

### Strengths and limitations

To our knowledge, this is the first study to take into account eating disorders as an effect modifier of the relationship between BMI and psychological health. Due to the population-based study design and comprehensive diagnostic procedures to identify eating disorders, the generalizability of our findings to young adults is good. The BMI and morbidity/mortality of adult twins do not differ from the general population [45-48]. Our study was cross-sectional, thus causality cannot be evaluated. BMI was a self-reported measure, yet the agreement between the measured and reported values was good [28]. Some of the instruments used in this study may be too crude to detect differences in the general population. This was most obvious for the measure of satisfaction with sex life, which was unable to detect differences between healthy women and women with a lifetime eating disorder, yet sexual dysfunction is common in women with eating disorders [49].

## Conclusions

Our study provides evidence for the inverse U-shaped relationship between BMI and subjective well-being in men. Categorization of body weight in terms of BMI may need to be reassessed in young men. To our knowledge, this is the first study to take into account eating disorders as potential effect modifiers of this relationship in women. Our findings indicate that eating disorders should be taken into consideration in future studies due to the strong inverse U-shaped relationship between BMI and subjective well-being in women with predisposition to such disorders.

## Abbreviations

AN: Anorexia nervosa; BN: Bulimia nervosa; BED: Binge-eating disorder

## Competing interests

The authors declare that they have no competing interests.

## Authors’ contributions

Author ML contributed to designing the current study, performed the statistical analysis of the data, and drafted the manuscript. Author JK conceived the FinnTwin16 study, contributed to FinnTwin16 wave 4 questionnaire, contributed to the current study design, data analysis, and interpretation of the data, and participated in drafting the paper. Authors ARa, ES, and AK-R contributed to the diagnostic interviews and revised the manuscript critically for important intellectual content. Authors AK-R and ARi contributed to FinnTwin wave 4 questionnaire, and ARi contributed to the current study design and interpretation of the data, and participated in drafting the paper. All authors read and approved the final manuscript.

## Pre-publication history

The pre-publication history for this paper can be accessed here:

http://www.biomedcentral.com/1471-2458/13/231/prepub

## Supplementary Material

Additional file 1: Figure S1 Distribution of BMI in men and in women. Click here for file

Additional file 2: Figure S2 The inverse U-shaped relationship between physical activity index and BMI (p<0.001) in men. Click here for file

## References

[B1] Must A Spadano J Coakley EH Field AE Colditz G Dietz WH The disease burden associated with overweight and obesity JAMA 1999 282 1523 1529 10.1001/jama.282.16.1523 10546691

[B2] Petry NM Barry D Pietrzak RH Wagner JA Overweight and obesity are associated with psychiatric disorders: results from the national epidemiologic survey on alcohol and related conditions Psychosom Med 2008 70 288 297 10.1097/PSY.0b013e3181651651 18378873

[B3] Luppino FS de Wit LM Bouvy PF Stijnen T Cuijpers P Penninx BW Overweight, obesity, and depression: a systematic review and meta-analysis of longitudinal studies Arch Gen Psychiatry 2010 67 220 229 10.1001/archgenpsychiatry.2010.2 20194822

[B4] Childers DK Allison DB The ‘obesity paradox’: a parsimonious explanation for relations among obesity, mortality rate and aging? Int J Obes 2010 34 1231 1238 10.1038/ijo.2010.71 PMC3186057 20440298

[B5] de Wit LM van Straten A van Herten M Penninx BW Cuijpers P Depression and body mass index, a U-shaped association BMC Publ Health 2009 9 14 10.1186/1471-2458-9-14 PMC2631467 19144098

[B6] Ford ES Moriarty DG Zack MM Mokdad AH Chapman DP Self-reported body mass index and health-related quality of life: findings from the behavioral risk factor surveillance system Obes Res 2001 9 21 31 10.1038/oby.2001.4 11346664

[B7] Wee HL Cheung YB Loke WC Tan CB Chow MH Li SC The association of body mass index with health-related quality of life: an exploratory study in a multiethnic Asian population Value Health 2008 11 Suppl 1 S105 S114 18387053 10.1111/j.1524-4733.2008.00374.x

[B8] Hopman WM Berger C Joseph L Barr SI Gao Y Prior JC The association between body mass index and health-related quality of life: data from CaM*os*, a stratified population study Qual Life Res 2007 16 1595 1603 10.1007/s11136-007-9273-6 17957495

[B9] Han TS Tijhuis MA Lean ME Seidell JC Quality of life in relation to overweight and body fat distribution Am J Public Health 1998 88 1814 1820 10.2105/AJPH.88.12.1814 9842379 PMC1509048

[B10] Fontaine KR Barofsky I Obesity and health-related quality of life Obes Rev 2001 2 173 182 10.1046/j.1467-789x.2001.00032.x 12120102

[B11] Larsson U Karlsson J Sullivan M Impact of overweight and obesity on health-related quality of life - a Swedish population study Int J Obes Relat Metab Disord 2002 26 417 424 10.1038/sj.ijo.0801919 11896499

[B12] Lean ME Han TS Seidell JC Impairment of health and quality of life in people with large waist circumference Lancet 1998 351 853 856 10.1016/S0140-6736(97)10004-6 9525361

[B13] de Wit L Luppino F van Straten A Penninx B Zitman F Cuijpers P Depression and obesity: a meta-analysis of community-based studies Psychiatry Res 2010 178 230 235 10.1016/j.psychres.2009.04.015 20462641

[B14] Diener E Subjective well-being Psychol Bull 1984 95 542 575 6399758

[B15] Ali SM Lindstrom M Socioeconomic, psychosocial, behavioural, and psychological determinants of BMI among young women: differing patterns for underweight and overweight/obesity Eur J Public Health 2006 16 325 331 16162598 10.1093/eurpub/cki187

[B16] McCrea RL Berger YG King MB Body mass index and common mental disorders: exploring the shape of the association and its moderation by age, gender and education Int J Obes 2011 36 414 421 10.1038/ijo.2011.65 21427699

[B17] Hudson JI Hiripi E Pope HG Jr Kessler RC The prevalence and correlates of eating disorders in the national comorbidity survey replication Biol Psychiatry 2007 61 348 358 10.1016/j.biopsych.2006.03.040 16815322 PMC1892232

[B18] Keski-Rahkonen A Hoek HW Susser ES Linna MS Sihvola E Raevuori A Epidemiology and course of anorexia nervosa in the community Am J Psychiatry 2007 164 1259 1265 10.1176/appi.ajp.2007.06081388 17671290

[B19] Keski-Rahkonen A Hoek HW Linna MS Raevuori A Sihvola E Bulik CM Incidence and outcomes of bulimia nervosa: a nationwide population-based study Psychol Med 2009 39 823 831 10.1017/S0033291708003942 18775085

[B20] Preti A Girolamo G Vilagut G Alonso J Graaf R Bruffaerts R The epidemiology of eating disorders in six european countries: results of the ESEMeD-WMH project J Psychiatr Res 2009 43 1125 1132 10.1016/j.jpsychires.2009.04.003 19427647

[B21] Keski-Rahkonen A Sihvola E Raevuori A Kaukoranta J Bulik CM Hoek HW Reliability of self-reported eating disorders: optimizing population screening Int J Eat Disord 2006 39 754 762 10.1002/eat.20277 16937380

[B22] First M Spitzer R Gibbon M Williams J Structured clinical interview for DSM-IV-TR axis I disorders, research version, non-patient edition (SCID-I/NP) 2002 New York: Biometrics Research, New York State Psychiatric Institute

[B23] Goldberg DP The detection of psychiatric illness by questionnaire 1972 London: Oxford University Press

[B24] Penninkilampi-Kerola V Miettunen J Ebeling H A comparative assessment of the factor structures and psychometric properties of the GHQ-12 and the GHQ-20 based on data from a Finnish population-based sample Scand J Psychol 2006 47 431 440 10.1111/j.1467-9450.2006.00551.x 16987212

[B25] Koivumaa-Honkanen H Kaprio J Honkanen R Viinamaki H Koskenvuo M Life satisfaction and depression in a 15-year follow-up of healthy adults Soc Psychiatry Psychiatr Epidemiol 2004 39 994 999 10.1007/s00127-004-0833-6 15583908

[B26] Koivumaa-Honkanen H Honkanen R Viinamaki H Heikkila K Kaprio J Koskenvuo M Life satisfaction and suicide: a 20-year follow-up study Am J Psychiatry 2001 158 433 439 10.1176/appi.ajp.158.3.433 11229985

[B27] Allardt E About dimension of welfare: an explanatory analysis of the comparative Scandinavian survey. University of Helsinki research group of comparative sociology research report 1 1973 Helsinki, Finland: University of Helsinki

[B28] Mustelin L Silventoinen K Pietilainen K Rissanen A Kaprio J Physical activity reduces the influence of genetic effects on BMI and waist circumference: a study in young adult twins Int J Obes 2009 33 29 36 10.1038/ijo.2008.258 19048013

[B29] Latvala A Dick DM Tuulio-Henriksson A Suvisaari J Viken RJ Rose RJ Genetic correlation and gene-environment interaction between alcohol problems and educational level in young adulthood J Stud Alcohol Drugs 2011 72 210 220 21388594 10.15288/jsad.2011.72.210 PMC3052891

[B30] Lahti-Koski M Pietinen P Heliovaara M Vartiainen E Associations of body mass index and obesity with physical activity, food choices, alcohol intake, and smoking in the 1982–1997 FINRISK studies Am J Clin Nutr 2002 75 809 817 11976153 10.1093/ajcn/75.5.809

[B31] Pajari M Pietilainen KH Kaprio J Rose RJ Saarni SE The effect of alcohol consumption on later obesity in early adulthood - a population-based longitudinal study Alcohol Alcohol 2010 45 173 179 10.1093/alcalc/agp090 20071348 PMC2842105

[B32] Blow FC Serras AM Barry KL Late-life depression and alcoholism Curr Psychiatry Rep 2007 9 14 19 10.1007/s11920-007-0004-z 17257508

[B33] Williams RL A note on robust variance estimation for cluster-correlated data Biometrics 2000 56 645 646 10.1111/j.0006-341X.2000.00645.x 10877330

[B34] Barry D Pietrzak RH Petry NM Gender differences in associations between body mass index and DSM-IV mood and anxiety disorders: results from the national epidemiologic survey on alcohol and related conditions Ann Epidemiol 2008 18 458 466 10.1016/j.annepidem.2007.12.009 18329894 PMC2504706

[B35] Zhao G Ford ES Dhingra S Li C Strine TW Mokdad AH Depression and anxiety among US adults: associations with body mass index Int J Obes 2009 33 257 266 10.1038/ijo.2008.268 19125163

[B36] Goldney RD Dunn KI Air TM Dal Grande E Taylor AW Relationships between body mass index, mental health, and suicidal ideation: population perspective using two methods Aust N Z J Psychiatry 2009 43 652 658 10.1080/00048670902970825 19530022

[B37] McLaren L Beck CA Patten SB Fick GH Adair CE The relationship between body mass index and mental health. A population-based study of the effects of the definition of mental health Soc Psychiatry Psychiatr Epidemiol 2008 43 63 71 10.1007/s00127-007-0269-x 17960318

[B38] Raevuori A Keski-Rahkonen A Bulik CM Rose RJ Rissanen A Kaprio J Muscle dissatisfaction in young adult men Clin Pract Epidemiol Ment Health 2006 4 2 6 16594989 10.1186/1745-0179-2-6 PMC1501012

[B39] Wee HL Wu Y Thumboo J Lee J Tai ES Association of body mass index with short-form 36 physical and mental component summary scores in a multiethnic Asian population Int J Obes 2010 34 1034 1043 10.1038/ijo.2010.24 20142824

[B40] Jia H Lubetkin EI The impact of obesity on health-related quality-of-life in the general adult US population J Public Health 2005 27 156 164 10.1093/pubmed/fdi025 15820993

[B41] Bjerkeset O Romundstad P Evans J Gunnell D Association of adult body mass index and height with anxiety, depression, and suicide in the general population: the HUNT study Am J Epidemiol 2008 167 193 202 17981889 10.1093/aje/kwm280

[B42] Afari N Noonan C Goldberg J Roy-Byrne P Schur E Golnari G Depression and obesity: do shared genes explain the relationship? Depress Anxiety 2010 27 799 806 10.1002/da.20704 20821799 PMC2949065

[B43] Lilenfeld LR Kaye WH Greeno CG Merikangas KR Plotnicov K Pollice C A controlled family study of anorexia nervosa and bulimia nervosa: psychiatric disorders in first-degree relatives and effects of proband comorbidity Arch Gen Psychiatry 1998 55 603 610 10.1001/archpsyc.55.7.603 9672050

[B44] Wade TD Bulik CM Neale M Kendler KS Anorexia nervosa and major depression: shared genetic and environmental risk factors Am J Psychiatry 2000 157 469 471 10.1176/appi.ajp.157.3.469 10698830

[B45] Rissanen A Heliovaara M Aromaa A Overweight and anthropometric changes in adulthood: a prospective study of 17,000 Finns Int J Obes 1988 12 391 401 3235260

[B46] Chitkara B Macdonald A Reveley AM Twin birth and adult psychiatric disorder. An examination of the case records of the Maudsley Hospital Br J Psychiatry 1988 152 391 398 10.1192/bjp.152.3.391 3167376

[B47] Kendler KS Martin NG Heath AC Eaves LJ Self-report psychiatric symptoms in twins and their nontwin relatives: are twins different? Am J Med Genet 1995 60 588 591 10.1002/ajmg.1320600622 8825903

[B48] Kaprio J The Finnish Twin cohort study: an update Twin Res Hum Genet 2013 epub ahead of print 10.1017/thg.2012.142 PMC4493754 23298696

[B49] Pinheiro AP Raney TJ Thornton LM Fichter MM Berrettini WH Goldman D Sexual functioning in women with eating disorders Int J Eat Disord 2010 43 123 129 19260036 10.1002/eat.20671 PMC2820601

