# Supplementary figures and images for: Body mass index and subjective well-being in young adults: a twin population study
Source: BMC Public Health. 2013 Mar 16;13:231. doi: 10.1186/1471-2458-13-231 (PMC3691623; doi:10.1186/1471-2458-13-231)

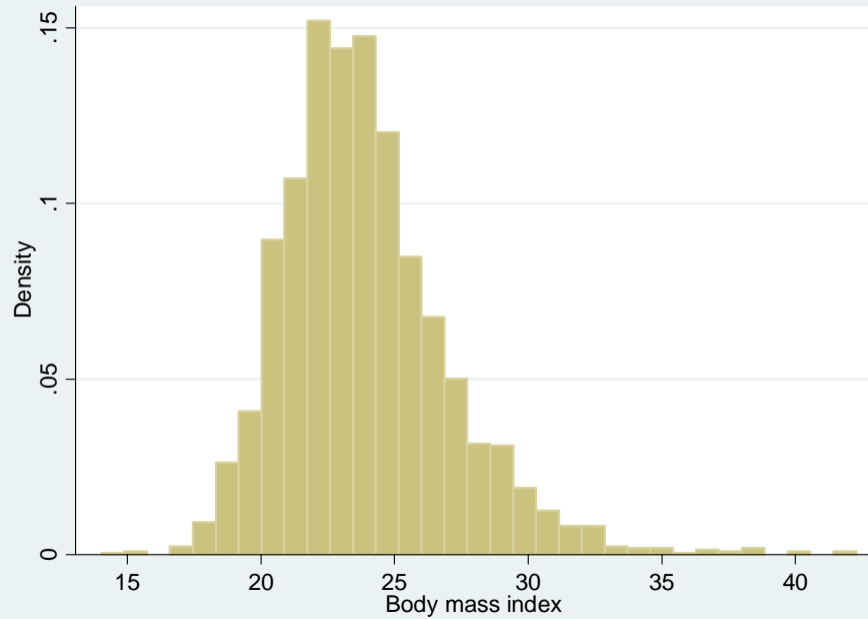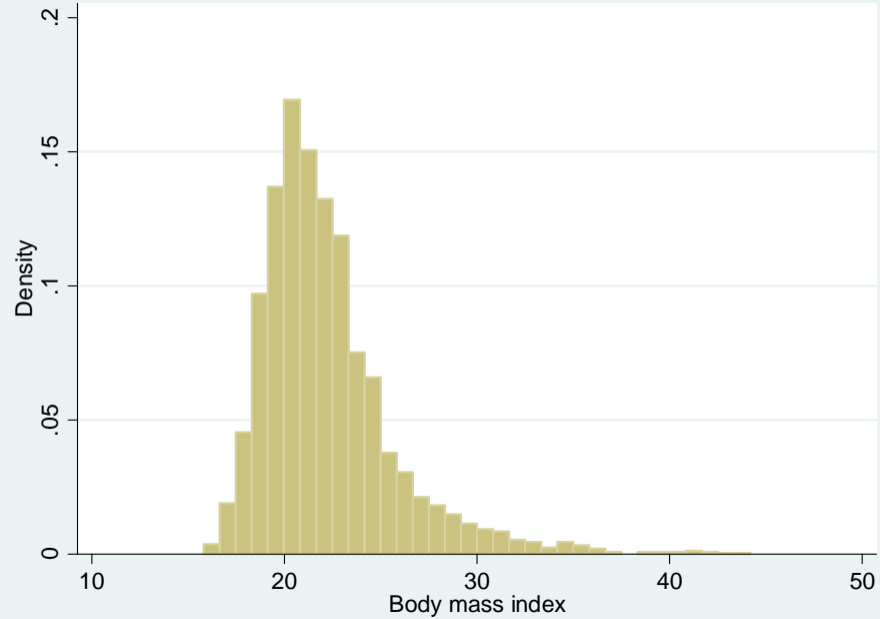

Supplement: Additional file 1: Figure S1 — Distribution of BMI in men and in women. [file 1471-2458-13-231-S1.pdf]

Physical activity index

15

10

5

0

15

20

25

30

35

40

Body mass index

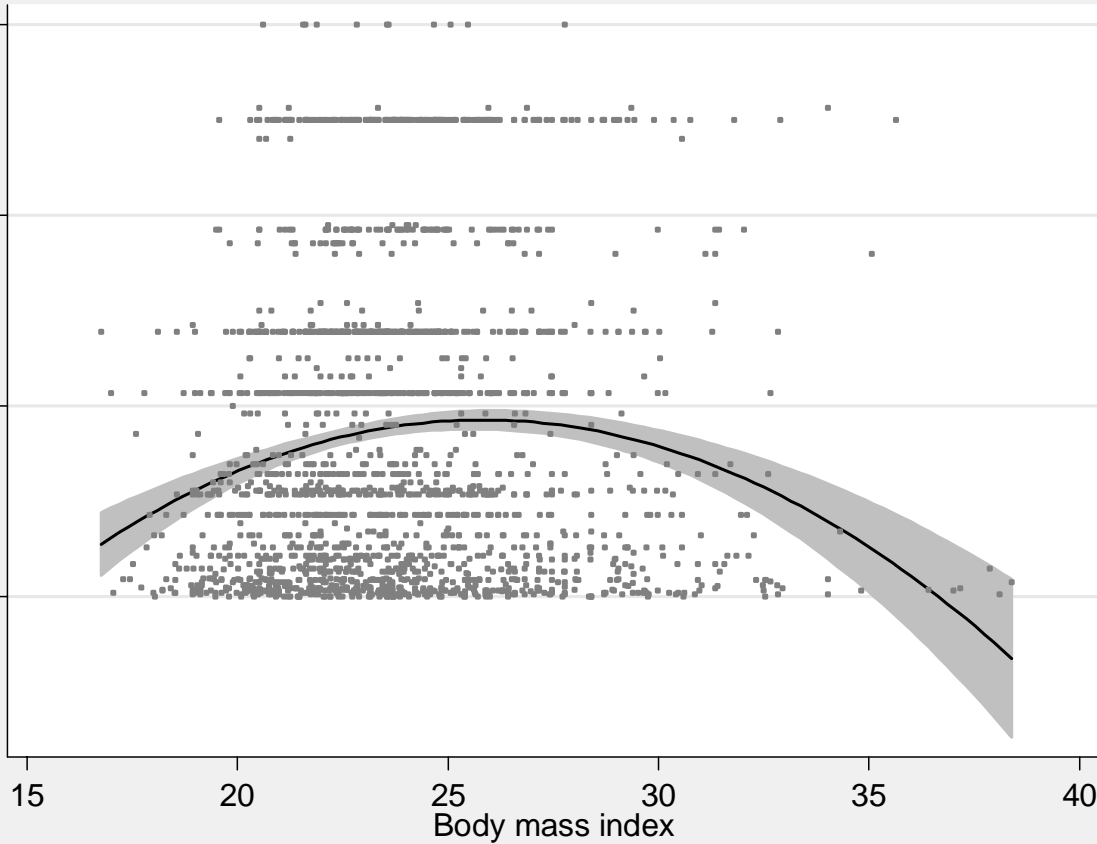

Supplement: Additional file 2: Figure S2 — The inverse U-shaped relationship between physical activity index and BMI (p<0.001) in men. [file 1471-2458-13-231-S2.pdf]
